# Exogenous TIPE2 Inhibit TAK1 to Improve Inflammation and Neuropathic Pain Induced by Sciatic Nerve Injury Through Inactivating NF-κB and JNK

**DOI:** 10.1007/s11064-022-03671-4

**Published:** 2022-07-16

**Authors:** Xuehua Sun, Xinyou Li, Youfei Zhou, Yufei Wang, Xiaochen Liu

**Affiliations:** Pain department, Yantai Affiliated Hospital of Binzhou Medical University, No. 717, Jinbu street, Muping District, Yantai City, 264100 Shandong People’s Republic of China

**Keywords:** TIPE2, TAK1, Sciatic nerve injury, NF-κB, JNK

## Abstract

Tumor necrosis factor-alpha-induced protein 8-like 2 (TIPE2) possesses potent anti-inflammatory effect. However, if TIPE2 ameliorates sciatic nerve injury (SNI)-induced inflammation and pain remains undiscussed, and the underlying role TAK1 in it were unknown. To verify our imagine, we performed SNI surgery, and analyzed expression and colocalization of TIPE2 and TAK1 in spinal cord and dorsal root neurons (DRG) by immunofluorescence staining and western blot. And the biological analysis, inflammatory factors, and pathological improvement were determined, and the regulation of TIPE2 in TAK1, phosphor-NF-κB, phospho-JNK was also tested by immunofluorescence staining and western blot. Experimental results showed the parabola-like change of TIPE2 and rising expression of TAK1 in spinal cord and DRG. And intrathecal TIPE2 injection could significantly improve the status of SNI rats, inhibit level of IL-6, IL-10 and TNF-α, raise the thermal withdrawal relax latency and mechanical withdrawal thresholds. Meanwhile, we also detected how TIPE2 regulated TAK1, and the downstream pathway NF-κB and JNK. The result indicated that TIPE2 could reduce TAK1 expression, and make NF-κB and JNK inactivated. To deeply discuss the potential mechanism, we injected TAK1 oligodeoxynucleotide into rats, and found that TIPE2 exerted the protective role against SNI through TAK1. In brief, TIPE2 reduced expression of TAK1, thereby inhibiting activation of NF-kB and JNK, further improving the neuroinflammation and neuropathic pain. TIPE2 played a protective role in sciatic nerve injury rats through regulating TAK1.

## Introduction

As an ordinary traumatic injury, sciatic nerve injury is caused by the injury of sciatic nerve trunk or branch, which could lead to innervating sensory and dysfunctional muscle disorder, painful feelings, and even permanent disability [1, 2]. Meanwhile, a series of secondary effects (such as inflammation, oxidative stress, and excitotoxicity) occurred, leading to aggravated damage [3, 4]. Previous studies have shown that in the inflammation of the central nervous system (CNS), inflammatory stimuli or injury-related factors activate astrocytes [5–7]. Besides, activated astrocytes in turn can further promote the neuroinflammatory response by releasing inflammatory cytokines and chemokines, which contributes to neuropathic pain [8]. After trauma injury of peripheral nerves, activated glial cells in the dorsal horn of spinal cord play a vital role in maintaining persistent pain states [9]. In model of chronic constriction of the sciatic nerve injury, increasing expression of GFAP in glial cell was found in the sciatic nerve and the dorsal root ganglion (DRG), and suppression of NF-κB in glial cells alleviated pain and inflammation after peripheral nerve injury [10].

As a member of the MAPK kinase family, TGF-beta-activated kinase 1 (TAK1) is found to boost the secretion of inflammatory factors [11]. p-TAK1 was highly expressed in sciatic nerve and DRG was found, and its antagonist markedly attenuated neuroinflammatory pain in rat with chronic constriction injury of the sciatic nerve [12]. Downregulation of TAK1 in spinal cord attenuates neuropathic pain [13]. Tumour necrosis factor (TNF)-alpha-induced protein 8-like 2 (TIPE2), a novel negative regulator of TAK1, blocks the TAK1-TAB1-TAB2 complex formation, further inhibits TAK1 activation and its downstream targets [14]. TIPE2 is reported to be a negative regulator of immunity and inflammation, maintaining immune homeostasis [15, 16], and it is also a crucial player in the inflammation-dependent diseases. Affymetrix gene chips were used to examine gene expression profiles, and 141 genes were found to be highly expressed in the inflamed spinal cord, one of which was TIPE2 [17]. Upregulation of TIPE2 inhibited microglial activation by inactivating Rac1/NF-κB, thereby lessening inflammatory pain [18]. Overexpression of TIPE2 reduces the levels of cytokines and inhibits inflammatory cell infiltration in cerebral ischemia–reperfusion [19]. However, whether TIPE2 regulate TAK1 to attenuate neuroinflammatory response and pain is unknown, and remains an unanswered question.

In this study, we constructed the rat model of sciatic nerve injury to explore the above innovative challenge. We investigated the expression of TIPE2 and TAK1 in spinal cord and DRG of animal model, and discussed if TIPE2 regulated TAK1 to mitigate inflammation and pain.

## Materials and Methods

### Animal and Experimental Design

Animal experiments were conducted in strict accordance to the principles of the Declaration of Helsinki. All experiments in this study were supported by the Institutional Animal Care and Use Committee of Yantai Affiliated Hospital of Binzhou Medical University. A total number of adult Sprague–Dawley (SD) rats (200–220 g, male) were provided from Jinan Pengyue Experimental Animal Breeding Co., Ltd (SYXK(Lu) 20180030). After acclimation for 7 days, sciatic nerve injury model was constructed.

In our experimental design, we firstly observed the expression of TIPE2 and TAK1 in posterior horn of spinal cord and DRG at different time points (3, 7, 14, 21 days), 4 rats in each time point. Then the rats were randomly divided into five groups (n = 8). (1) Sham group: the rats received the same procedures except for compression. (2) SNI group: the rats received the SNI procedures, then after 3 days, the rats were treated with the 25 μL saline water. (3) TIPE2-1 μg/kg group: 3 days after SNI surgery, the rats were injected 25 μL 1 μg/kg recombinant human TIPE2 protein (rhTIPE2, ab164054, Abcam) into L5 vertebrae. (4) TIPE2-5 μg/kg group: SNI surgery is followed by rhTIPE2 treatment at a dose of 5 μg/kg. (5) TIPE2- 5 + TAK1ODN group: SNI rats were treated successively with 5 μg/kg rhTIPE2 and 0.5 nmol/μL [13] TAK1 oligodeoxynucleotide (ODN). The sequence of ODN of TAK1 was 5′-GCACTCCCTTTATTATTG-3′ (Genepharma). All administration was given by the intrathecal delivery [13, 20] every 2 days for six consecutive times. After the experiment, the rats were injected 3% pentobarbital sodium (50 mg/kg) into abdominal, then sacrificed by a cervical dislocation. The L5 spinal cord segments and DRG were collected for pathology experiment and western blot.

### Sciatic Nerve Injury Model

According to the previously reported method, we constructed the sciatic nerve injury (SNI) model [21]. In short, the rats were anesthetized by intraperitoneal injection of 3% pentobarbital sodium (50 mg/kg). Expose the right sciatic nerves, then compress it for 3 times (10 s per time) by a forceps at approximately 5 mm away from the bifurcation, then suture the skin, and place rats into the original feeding environment. The rats in sham group received the same procedures except compression.

### Behavioral Detection

Hot plate test and mechanical withdrawal thresholds were ordinarily used to evaluate the sensory function recovery of SNI rats on preoperative day 1 and postoperative days 3, 5, 7, 9, 11, 14, 17 and 21 according to the previous methods [22]. Hot plate test: concisely, the hind paw of rat stood on the hot plate (56 ℃). The time from contacting heat plate to departure was recorded, and the result is expressed as thermal withdrawal relax latency (TWRL). At 5-min intervals, the rats need to perform experiments in quadruplicate. It should be noted that when the retention time of hind paw on hot plate exceeded 12 s, TWRL value was recorded as 12 s. Mechanical withdrawal thresholds: The von Frey filaments were exploited to measure the paw withdrawal threshold after SNI with the range of force from 1 to 50 g within 20 s at the force speed of 2.5 g/s, and held continuously for 10 s when the force reached 50 g. After adapting to a plastic cage with a metal mesh floor, von Frey filaments were used to contact the midfoot surface of the left hind paw for 6–8 s. Abrupt withdrawal was considered as positive responses. Left hind paw was measured for at least three times, and the mean values were calculated.

#### ELISA

Prior to orbit blood collection, rats were fasted for 24 h. Then the blood was naturally solidified and centrifuged at 3000 rpm for 20 min. Finally, the liquid supernatant was collected for enzyme-linked immunosorbent assay (ELISA) analysis. The kits of IL-6 (H007-1-1), IL-10 (H009-1) and TNF-α (H052-1) were purchased from Nanjing Jiancheng Bioengineering Institute (China, Nanjing).

#### HE Staining

Spinal cord tissues and DRG tissues were fixed in 4% paraformaldehyde for 48 h, then embedded in paraffin. 5 μm-thick sections were dewaxed and rehydrated. Then the slices were stained with hematoxylin and eosin. Then, gradient dehydration, ditoluene transparent and neutral resin seal were carried out. Finally, the pathological changes of tissue sections were observed under microscope (LEICA DM 1000 LED). Then, we also quantified the severity of immune response in spinal cord and DRG. Score 0, no immune response; score 1, mild immune response; score 2, severe immune response; score 3, very severe inflammatory reaction.

#### Immunofluorescence Staining

After dewaxing and rehydration, 5 μm of paraffin sections were incubated with endogenous peroxidase at room temperature for 20 min, followed by antigen repair. Then the slices were incubated with 5% normal goat serum for 30 min, and went on incubating with diluted primary antibodies in 4 ℃ overnight. Next, the second antibodies were added to the slices, and cultured continuously at room temperature for 1 h. DAPI solution (C1005, Beyotime) was used to stained the cell nucleus for 10 min. Finally, the sections were sealed by resinene. The pictures of tissues were photoed using confocal microscopy (LSM800, Zeiss, German). Image J 1.49p was used to analyze the mean fluorescence intensity of TIPE2 and TAK1 in spinal cord and DRG. The detailed information of antibodies were as followed: (1) primary antibodies: rabbit anti-rat TIPE2 polyclonal antibody (1:550, 15940-1-AP, Proteintech), rabbit anti-rat TAK1 polyclonal antibody (1:80, bs-3585R, Bioss), mouse anti-rat TAK1 antibody (1:250, 67707-1, proteintech), rabbit anti-rat p-JNK1 + JNK2 (phospho T183 + Y185) (1:150, ab131499, Abcam), mouse anti-rat GFAP monoclonal antibody (1:600, 60190-1-lg, Proteintech); (2) second antibodies: goat anti-rabbit Cy3-labeled IgG (H + L) (1:500, A0516, Beyotime), goat anti-mouse FITC-labeled IgG (H + L) (1:500, A0568, Beyotime).

#### Western Blot

Spinal cord tissues were homogenized, and total protein was extracted using RIPA lysis, followed by quantitative analysis by bicinchoninic acid (BCA) protein assay kit. 40 μg samples were separated by 10% SDS-PAGE, and then transported to PVDF membranes. After blocking by 5% non-fat milk, the membranes were incubated with primary antibodies against p-IκBα (1:10000, ab133462, Abcam), IκBα (1:800, ab95338, Abcam), p-JNK1(1:1200, ab47337, Abcam), p-JNK2 (1:1000, P02706, Boster), JNK1 (1:2500, ab199380, Abcam), JNK2 (1:1500, 51153-1-AP, Proteintech), β-actin (1:2000, 20536-1-AP, Proteintech), TIPE2 (1:1000, orb158628, Biorbyt), TAK1 (1:800, orb256677, Biorbyt) at 4 ℃ overnight. After continuous incubation with secondary antibody goat anti-rabbit IgG H&L (HRP) (1:3000, ab6721, Abcam) for 1 h, protein bands were observed with enhanced chemiluminescence (ECL) solution. Quantitative result of band was analyzed by Image J 1.49p.

### Statistical Analysis

In this study, SPSS version 19.0 was used to perform the statistical analysis. The data in all statistical maps was represented as mean ± standard deviation (SD). Statistical difference in more than or equal to three groups was analyzed by one-way analysis of variance (ANOVA) followed by Tukey’s post-test. p Was more than 0.05, indicating the significant difference between multiple groups.

## Results

### The Expression of TIPE2 and TAK1 in Spinal Cord and DRG Tissues at the Different Time Points After SNI

**Fig. 1:**
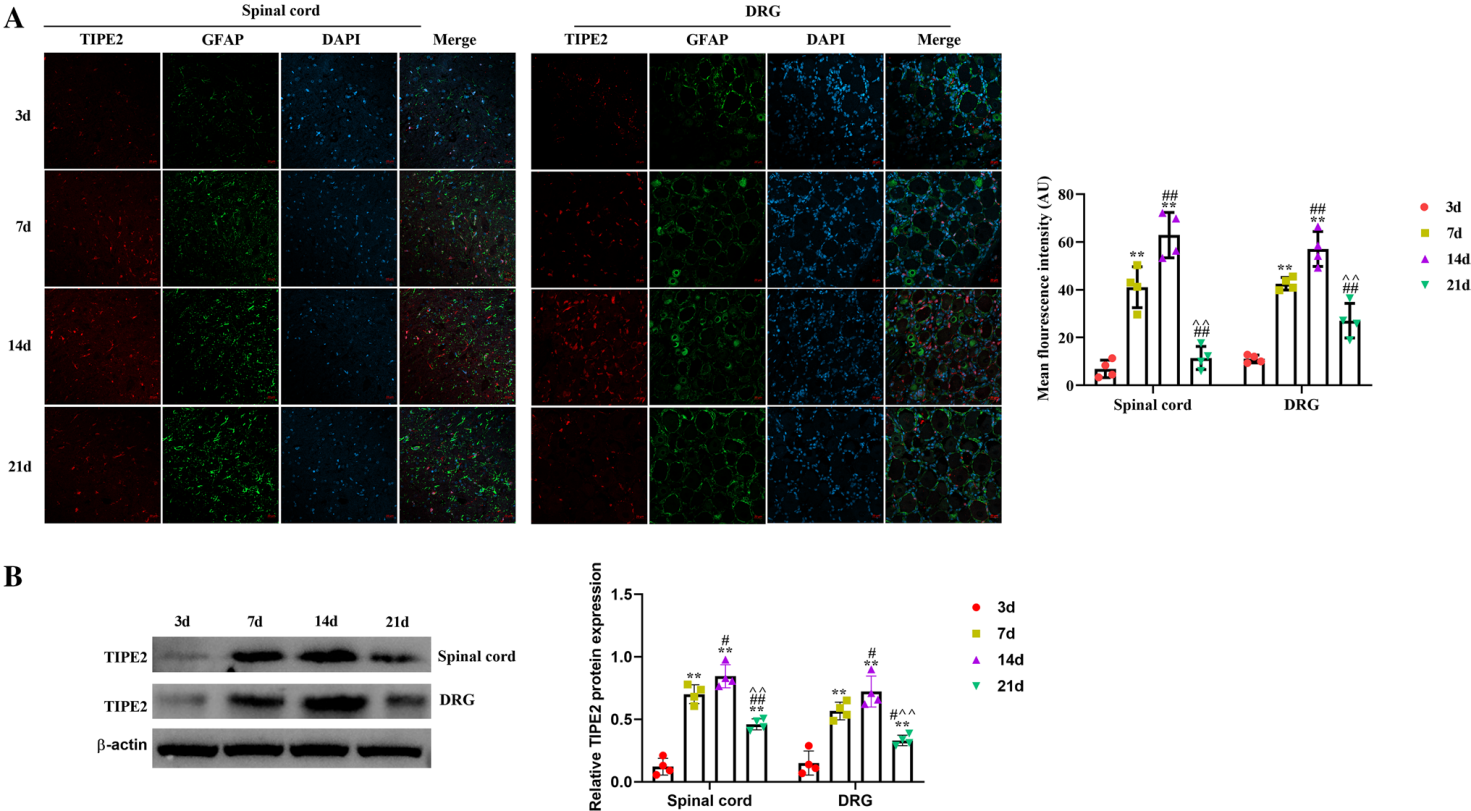
The expression of TIPE2 in spinal cord and DRG at different time points (3 days, 7 days, 14 days and 21 days), which was measured by double-staining immunofluorescence (**A**) and western blot (**B**). The quantified result was showed as mean fluorescence intensity, analyzing by Image J 1.49p. ^**^p < 0.01 versus 3 days; ^##^p < 0.01 versus 7d; ^^^^p < 0.01 versus 14 days. The scale bar of CLSM images is 20 μm. n = 4

**Fig. 2:**
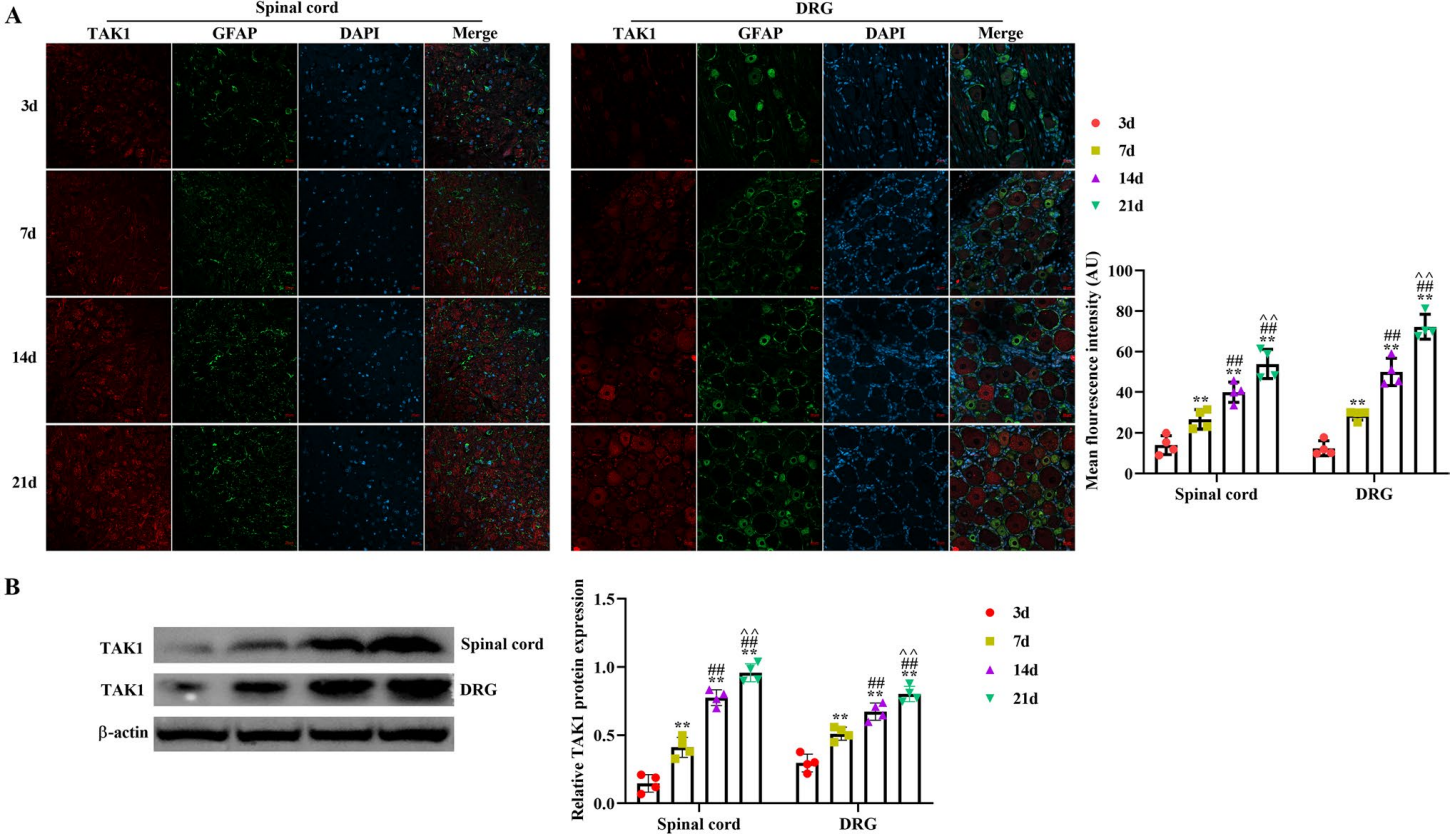
The expression of TAK1 in spinal cord and DRG at different time points (3 days, 7 days, 14 days and 21 days) was assayed by double-staining immunofluorescence (**A**) and western blot (**B**). TAK1-positive result was presented as mean fluorescence intensity in the bar graph, analyzing by Image J 1.49p software. The scale bar of the images is 20 μm. ^**^p < 0.01 versus 3d; ^##^p < 0.01 versus 7 days; ^^^^p < 0.01 versus 14 days. The scale bar of CLSM images is 20 μm. n = 4

**Fig. 3:**
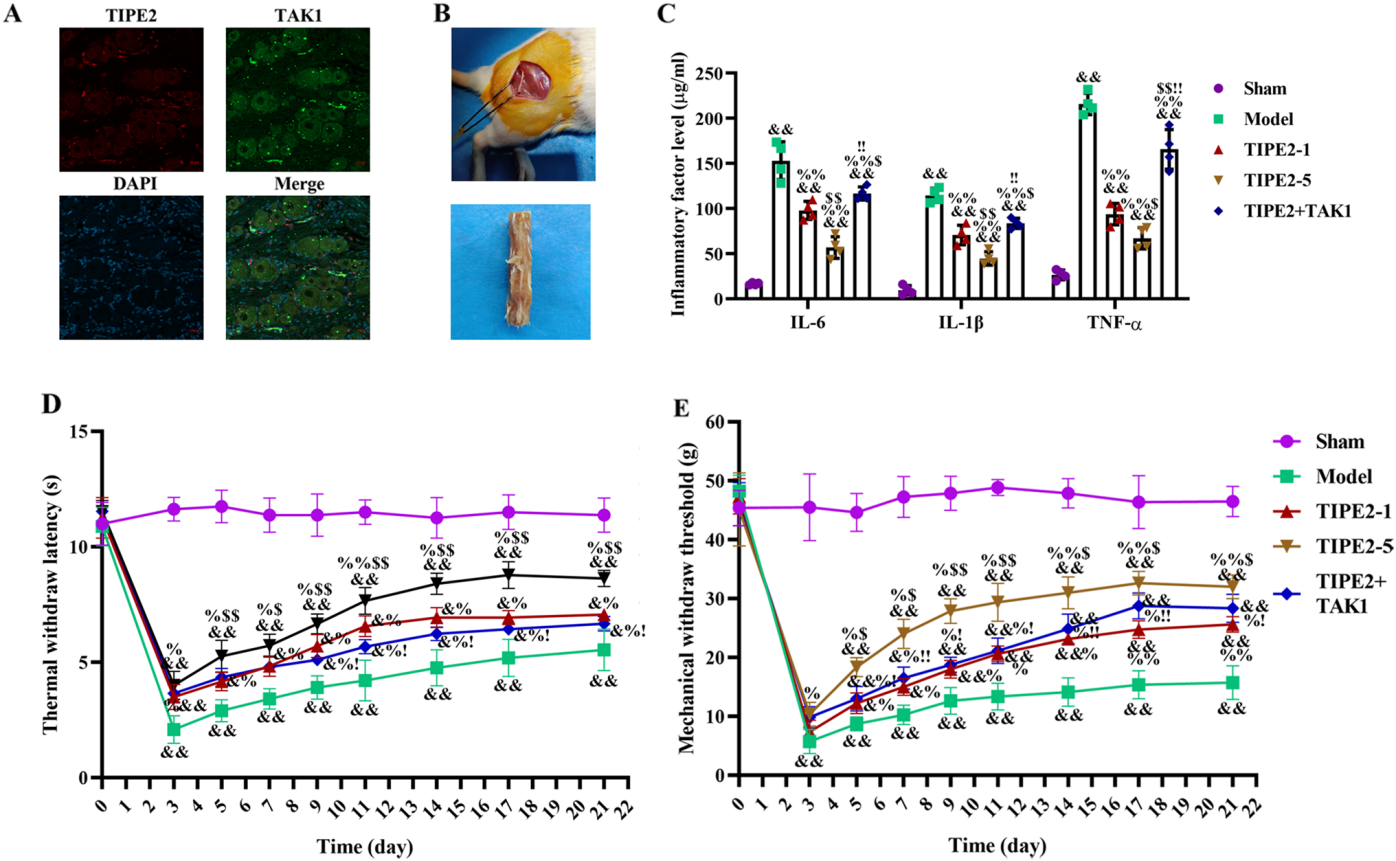
TIPE2 improved neuroinflammation and neuropathic pain in SNI rats. **A** Colocalization of TIPE2 (red) and TAK1 (green) in DRG. Scale bar of CLSM images was 20 μm. **B** The images of sciatic nerve injury model. **C** Levels of IL-6, IL-1β and TNF-α were measured by ELISA at the ending of experiment. **D** Hot plate test and mechanical withdrawal thresholds (**E**) were used to evaluate the neuropathic pain for 21 days. ^&^p < 0.05, ^&&^p < 0.01 versus Sham; ^%^p < 0.05, ^%%^p < 0.01 versus Model; ^$^p < 0.05, ^$$^p < 0.01 versus TIPE2-1; ^!^p < 0.05, ^!!^p < 0.01 versus TIPE2-5. n = 4

Firstly, immunofluorescence staining and western blot were used to assess localization and content of TIPE2 (Fig. 1) and TAK1 (Fig. 2) in two tissues. For TIPE2 in spinal cord and DRG tissues, there was the parabola-like trend in a time-dependent manner. The mean fluorescence intensity of TIPE2 reached a peak level at 14 days, then showed a declined trend. After 21 days, the intensity of TIPE2 was closed to that of 3 days. There was no significant difference between 3 and 21 days (p > 0.05). There was a potent difference between 3 and 7 days, 3 days and 14 days, 7 days and 14 days, 7 days and 21 days, 14 days and 21 days (p < 0.01). Meanwhile, TAK1-positive staining results showed a rising tendency accompanied by an extension of time. And the highest expression of TAK1 was detected at 21d. There was prominent otherness between each time point (p < 0.01). Consistent with the immunofluorescence staining, western blot of TIPE2 and TAK1 proteins also indicated the similar trend. We also detected the colocalization of TAK1 and TIPE2 in DRG (Fig. 3A). TAK1 were abundant in neurons and TIPE2 was mainly expressed in satellite glial cells.

### The Behavioral Evaluation and Inflammatory Factors Affected by Administration of TIPE2 Through TAK1

The images of sciatic nerve injury model were shown in Fig. 3B. Meanwhile, ELISA method was applied to measured the level of inflammatory factors IL-6, IL-1β and TNF-a in serum (Fig. 3C). And the result showed that the level of IL-6, IL-1β and TNF-a in Model group was higher than that of sham group (p < 0.01), while the declined content occurred after TIPE2, especially in TIPE2-5 group. To further validate the role of TAK1 as the downstream mediator of TIPE2, TAK1 was intrathecally injected into L5 vertebrae of rat in TIPE2-5 group. We found an ascendant level of proinflammatory factors after delivery of TAK1, significantly higher than TIPE2-5 groups (p < 0.01). Additionally, we continued to perform the behavioral experiment to evaluate the sensory function recovery of SNI rats: Hot plate test (Fig. 3D) and mechanical withdrawal thresholds (Fig. 3E). Sciatic nerve injury caused the neuropathic pain with a notable reduction of mechanical withdrawal thresholds and thermal withdraw latency which were lower than those of sham group (p < 0.01). Rats in TIPE2 group showed an increase in mechanical withdrawal thresholds and thermal withdraw latency, and higher dose of TIPE2 had a more significant promoting effect compared to SNI rats. But the mechanical thresholds and thermal hyperalgesia in TIPE2 + TAK1 group were significantly less compared to TIPE2-5 group and higher compared to model group. These results indicated that TIPE2 could enhance mechanical and thermal hyperalgesia of SNI rats, which may have a close relationship to TAK1.

### TIPE2 Improved Damaged Spinal Cord and DRG Tissues

**Fig. 4:**
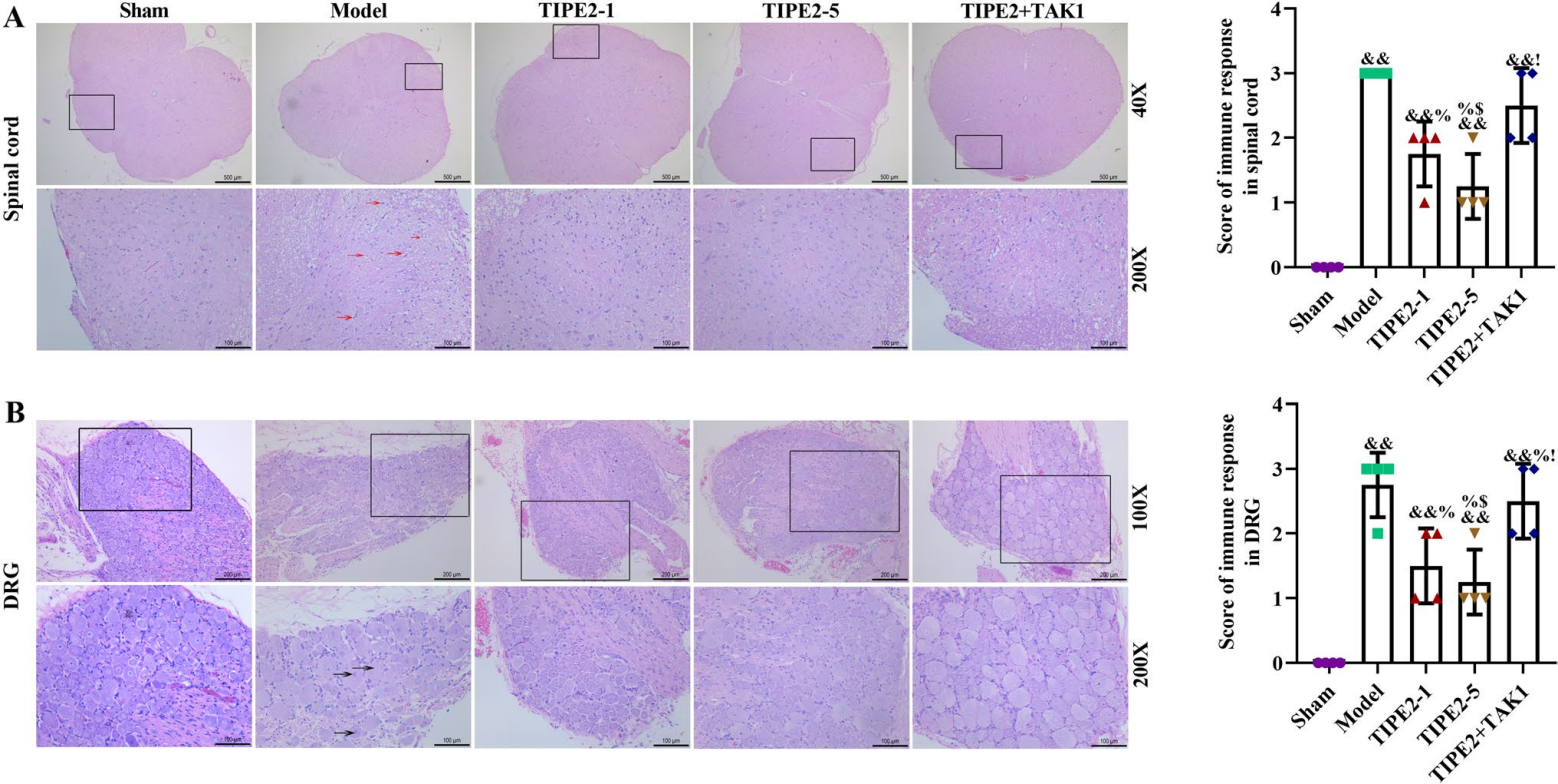
TIPE2 improved damaged spinal cord (**A**) and DRG tissues (**B**). After 14 days, spinal dorsal horn and DRG were collected and fixed, HE staining was used to observe the pathological damage. Spinal cord: scale gauges were 500 μm and 100 μm; DRG: scale gauges were 200 μm and 100 μm. Red arrows indicated the damaged neuron cells in spinal cord. Black arrows indicated the damaged neuron cells in DRG tissues. Score of immune response in spinal cord and DRG tissues was quantified to evaluate the severity of immune response. n = 4

HE staining was also used to visualize pathological damage in spinal cord and DRG. HE staining of spinal cord (Fig. 4A) and DRG (Fig. 4B) demonstrated an outstanding improvement of TIPE2. In model group, we observed disordered structure of posterior horn of spinal cord tissues and inflammatory cells’ infiltration, decreased number of neuronal cells. After treatment of TIPE2, the disrupted structure of spinal cord tissues was improved, especially 5 μg/kg TIPE2. However, TAK1 could aggravate the pathological injury of spinal cord tissues in posterior horn of spinal cord. For pathological change of DRG, we found a complete and normal structure of normal DRG tissues. Sciatic nerve injury could lead to damaged DRG with increasing intercellular space, some nuclei disappeared. After injection of TIPE2, the above histopathological changes were significantly improved. But TAK1 could counteract the positive effect of TIPE2, and even aggravated the DRG injury. Quantitative scores of spinal cords and DRG were also evaluated according to the immune response of each slice. Model group has the highest inflammation score, but TIPE2 could ameliorate the immune inflammation with a lower score, particularly 5 μg/kg TIPE2. But score in TIPE2 + TAK1 group was lower than model group, and higher than TIPE2 group.

### TIPE2 Inhibited TAK1 in Spinal Cord and DRG Tissues of SNI Rats

**Fig. 5:**
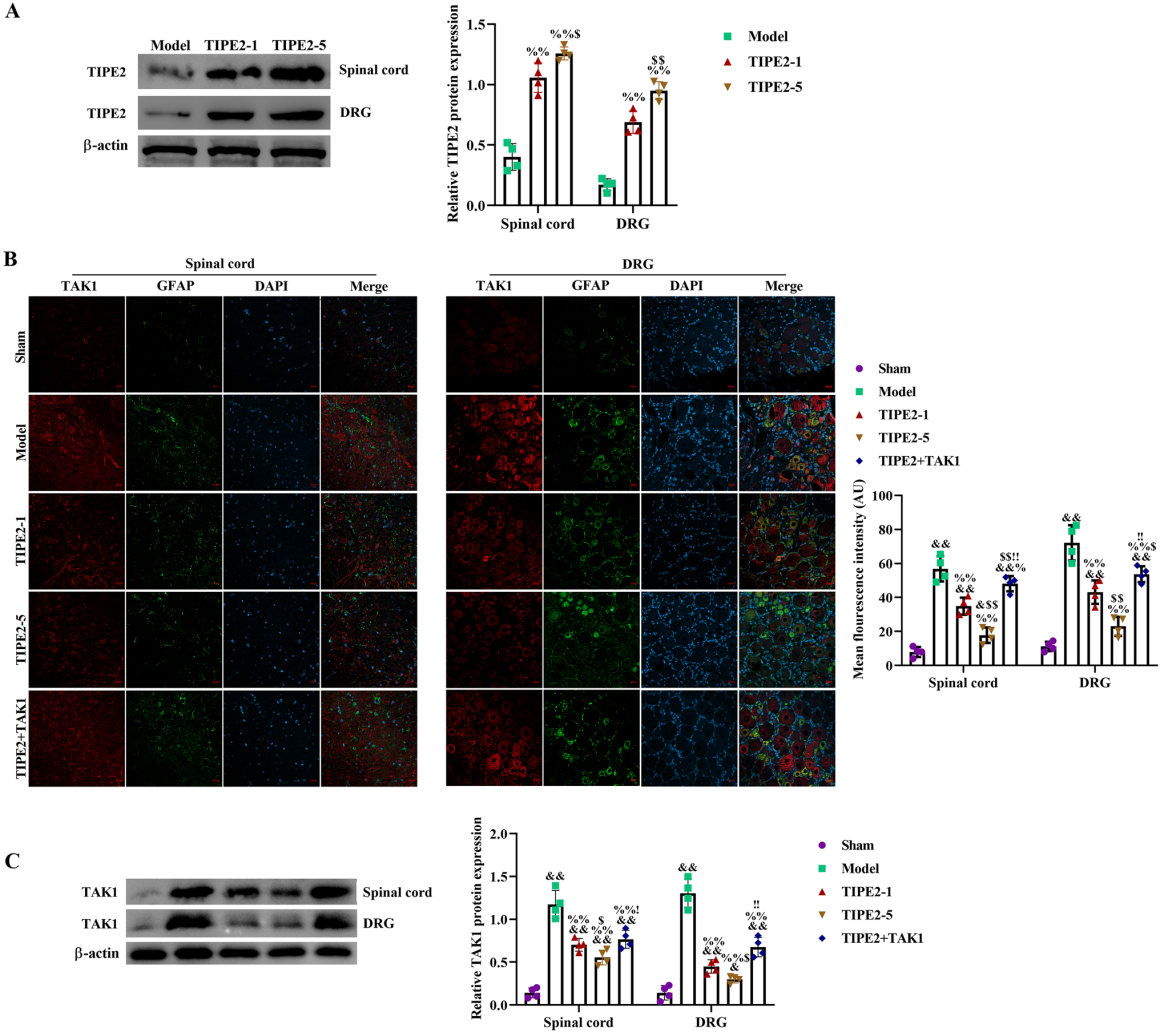
After SNI, administration of different-doseTIPE2 reduced the positive intensity of TAK1 in a dose-dependent manner. **A** After TIPE2 administration for 6 times, TIPE2 expression in spinal cord and DRG was measured by western blot. TAK1 expression was measured by double-staining immunofluorescence **B** and western blot (**C**). The mean fluorescence intensity of TAK1 in spinal cord and DRG was analyzed by Image J 1.49p. ^&^p < 0.05, ^&&^p < 0.01 versus Sham; ^%^p < 0.05, ^%%^p < 0.01 versus Model; ^$^p < 0.05, ^$$^p < 0.01 versus TIPE2-1; ^!!^p < 0.01 versus TIPE2-5. The scale bar of CLSM images is 20 μm. n = 4

The regulation of TIPE2 in TAK1 was explored and tested by immunofluorescence experiments (Fig. 5). First, we tested the expression of TIPE2 in spinal cord and DRG after TIPE2 injection by western blot (Fig. 5A). The expression of TIPE2 in two TIPE2 groups was significantly raised compared with model group, and there was a higher expression in TIPE2-5 group. Second, we found an enhancive fluorescence intensity of TAK1 in spinal dorsal horn and DRG, which could be inhibited by rhTIPE2 injection. A significantly decreasing appearance of TAK1-positive expression could be observed in TIPE2-5 group compared to TIPE2-1 group (p < 0.01). The interaction between TIPE2 and TAK1 was deeply discussed through addition of TAK1, indicating the regulation of TIPE2 in TAK1 (Fig. 5B). W also used western blot to measured its expression (Fig. 5C). Consistent with results immunological staining, western blot showed a same trend of TAK1 protein.

### TIPE2 Inhibited Activation of NF-κB and JNK via Downregulation of TAK1

**Fig. 6:**
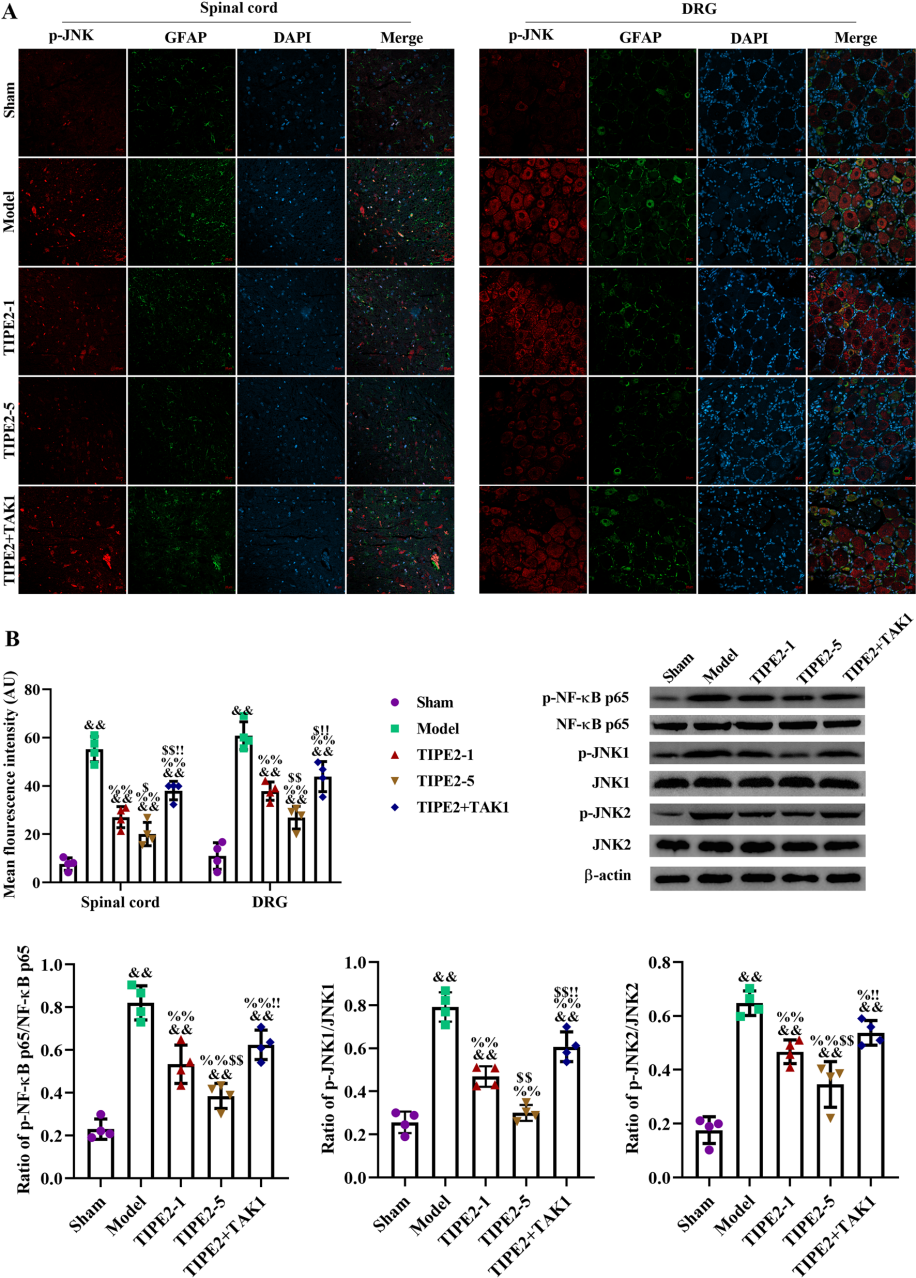
TIPE2 inhibited activation of NF-κB and JNK via downregulation of TAK1. **A** Immunofluorescence staining was performed to analyze phosphor-JNK in spinal cord and DRG after 14 days. Mean fluorescence intensity of phospho-JNK was analyzed by Image J 1.49p. The scale bar was 20 μm. **B** Western blot was carried out to analyze the expression of p-NF-κB p65, p-JNK1, and p-JNK2 in spinal cord after 14 days. ^&&^p < 0.01 versus Sham; ^%^p < 0.05, ^%%^p < 0.01 versus Model; ^$^p < 0.05, ^$$^p < 0.01 versus TIPE2-1; ^!^ p < 0.05, ^!!^p < 0.01 versus TIPE2-5

In this study, we used immunofluorescence assay and western blot to analyze phosphor-JNK. We found activated JNK in spinal cord and DRG of SNI rats with a higher expression compared to sham group (p < 0.01). After two doses of TIPE2, phosphorylation of JNK was inhibited. There was significant difference between model group and TIPE2-1/TIPE2-5 (p < 0.01). So how TIPE2 regulate phospho-JNK, and if it is related to TAK1 were unknown. Therefore, we used the TAK1- oligodeoxynucleotide to confirm those problems. A significant increase of p-JNK occurred after treatment of TIPE2-5 and TAK1, which is higher than TIPE2-5 group (p < 0.01) and lower than model group (p < 0.01). Phospho-JNK1 and phospho-JNK2 were detected by western blot, and the results were consistent with immunofluorescence staining. After that, we also analyzed if NF-κB p65 was activated by western blot. We also found a lower expression of p-NF-κB p65 caused by TIPE2. We also dissected the role TAK1 in inactivated NF-κB p65 induced by TIPE2. Finally, we found that TIPE2 decreased the expression of phospho-JNK1, phosphor-JNK2 and p-NF-κB p65, and the potential mechanism is likely to be related to TAK1 (see Fig. 6).

## Discussion

In the present design, the gist of our experiments are as follows: (1) TIPE2 exhibited the dynamic trend to increase first and then decrease in the spinal cord and DRG of SNI model; (2) Intrathecal injection of TIPE2 could decrease the GFAP expression, altering the number of astrocytes. (3) External administration of TIPE2 could enhance the functional recovery and reduce inflammation of sciatic nerve injury through downregulation of TAK1, which inactivated NF-κB. To sum up, it is speculated that activated astrocytes in sciatic nerve injury could aggravate the inflammation response and neuropathic pain. TIPE2 alter the increased expression of pro-inflammatory mediators IL-1β, IL-6 and TNF-α, and reduce pain sensation. TAK1 was highly expressed in damaged spinal cord and DRG, which is the potential downstream regulator of TIPE2. In our study, we verified descending TAK1 by TIPE2 is beneficial to damage repair of SNI rats. Therefore, we successfully validated that TIPE2 reduced expression of TAK1, thereby inhibiting NF-kB activation to play a protective role in sciatic nerve injury rats.

**Fig. 7:**
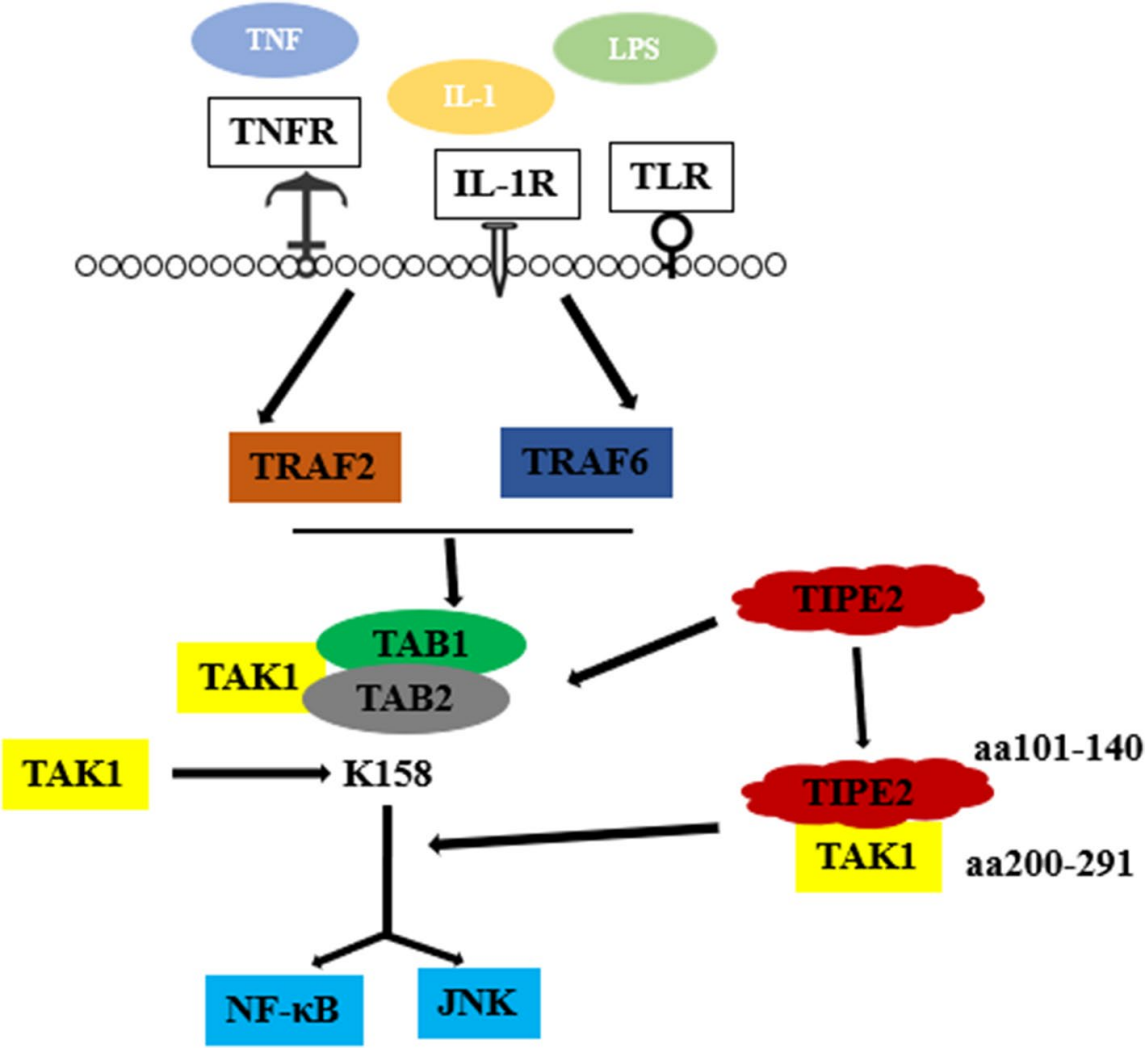
A possible process of TIPE2 inhibition of activation of TAK1 signaling. Binding between aa101-104 region on TIPE2 and aa 200-291 region of the internal kinase domain of TAK1 blocked the formation of TAK1-TAK1-binding protein (TAB) 1-TAB2 complex, further to interfer TAK1 activation. Therefore, the negative regulation by TIPE2 of TAK1 activation may lead to inhibition of TAK1-NF-κB mediated inflammatory responses

A previously reported study has demonstrated the interaction between TIPE2 and TAK1 in RAW264.7 cells and primary cells separated from spleen and thymus [14]. As shown in Fig. 7, the binding between aa101-104 region on TIPE2 and aa 200-291 region of the internal kinase domain of TAK1 blocked the formation of TAK1-TAK1-binding protein (TAB) 1-TAB2 complex, further to interfer TAK1 activation and abnormal expression of its downstream molecules. We took advantage of immunofluorescence staining to analyze the FITC-labeled TAK1, and found a descending intensity of TAK1 after treatment of recombinant human TIPE2 antibody. The result told us the potential regulatory role of TIPE2 on TAK1 in damaged spinal cord and DRG of SNI model. In addition, we also found the altered expression of NF-κB, which is likely due to abnormal TAK1. TAK1 activation by microbial mediators (such as lipopolysaccharide (LPS), TNF-α, and IL-1, et al.) is followed by phosphorylation of IKK complex, ultimately leading to NF-kB activation [23–25]. Therefore, NF-κB pathway is the conceivable downstream pathway of TAK1 in rats suffered with SNI.

Meanwhile, we also explored the phosphorylated degree of c-Jun NH(2)-terminal kinases (JNK) through immunofluorescence and western blot assays, and then found the increasing content of phosphor-JNK in model group, while TIPE2 could inhibit its activation of JNK. Besides we also add the ODN of TAK1 to confirm that the inhibition of JNK by TIPE2 acts through TAK1. TAK1, one of the the MAPK kinase kinase (MAP3K) family, acts on the activation of MAPK activation mediated by TGF-β [26–29]. TAK1 is absolutely requisite for TGF-β-induced JNK and NF-κB activation [30]. Experimental verification illustrated that TIPE2 can act on JNK by TAK1.

Actually, there are several points in this design that need to be further studied. (1) The activation of other inflammatory cells including microglia and macrophage also need to be discussed, and how TIPE2 regulates this activation will be validated in SNI model. (2) The protective effect of TIPE2 on compressed sciatic nerve, and how to promote the regeneration and interrupt degeneration of sciatic nerve, and if the insidious mechanisms of TIPE2 on sciatic nerve was related to TAK1. All of above problems deserve our further discussion.

## Data Availability

The datasets used and/or analyzed during the current study are available from the corresponding author on reasonable request.
